# Intestinal Permeability Is Associated with Mortality and Readmission in Children Hospitalized with Severe Acute Malnutrition

**DOI:** 10.1016/j.tjnut.2026.101655

**Published:** 2026-06-06

**Authors:** Jonathan P Sturgeon, Bernard Chasekwa, Joice Tome, Cherlynn Dumbura, Florence D Majo, Deophine Ngosa, Kuda Mutasa, Ellen Besa, Kusum J Nathoo, Claire D Bourke, Tafhima Haider, Robert Ntozini, Mutsa Bwakura-Dangarembizi, Beatrice Amadi, Paul Kelly, Andrew J Prendergast

**Affiliations:** 1 Zvitambo Institute for Maternal and Child Health Research, Harare, Zimbabwe; 2 Centre for Genomics and Child Health, Blizard Institute, Queen Mary University of London, London, United Kingdom; 3 Department of Infectious Disease, Section of Paediatric Infectious Disease, Faculty of Medicine, Imperial College London, United Kingdom; 4 Tropical Gastroenterology and Nutrition Group, University of Zambia, Lusaka, Zambia; 5 Department of Paediatrics and Child Health, Faculty of Medicine and Health Sciences, University of Zimbabwe, Harare, Zimbabwe

**Keywords:** severe acute malnutrition, intestinal permeability, LPS, malnutrition enteropathy, systemic inflammation

## Abstract

**Background:**

Severe acute malnutrition (SAM) is the most critical form of undernutrition, associated with high inpatient and postdischarge mortality. It is hypothesized that malnutrition enteropathy contributes to systemic inflammation by allowing translocation of intestinal pathogen–associated molecular patterns across the damaged epithelium.

**Objectives:**

Assess intestinal permeability, markers of bacterial translocation, and correlate these with inflammatory markers in children admitted with SAM and during 1 y after discharge.

**Methods:**

In a substudy of the longitudinal Health Outcomes, Pathogenesis and Epidemiology of Severe Acute Malnutrition observational study, we recruited 264 children with complicated SAM on admission to hospital at 3 centers in Zimbabwe and Zambia. We measured gut permeability by urinary lactulose:mannitol ratio (LMR); plasma lipopolysaccharide (LPS) using the limulus amoebocyte lysate assay; and plasma inflammatory biomarkers by enzyme-linked immunosorbent assay and Luminex, at admission, discharge, 12- and 48-wk postdischarge. Values were compared with 173 adequately nourished community controls. Data were analyzed using mixed effects models for longitudinal data, regression for associations between groups, and Cox proportional hazards models for survival.

**Results:**

Gut permeability, as measured by LMR, was 2.5-fold higher in children with SAM compared with community controls [95% confidence interval (CI): 1.4, 4.5], and only resolved by 48-wk postdischarge. Higher inpatient gut permeability was associated with increased risk of death or readmission over the following year [hazard ratio 6.14 (95% CI: 1.03, 36.8) per 10-fold increase in LMR]. Children with high gut permeability had higher levels of the intestinal biomarker glucagon-like peptide-2 and lower L-selectin. Children with higher plasma LPS had independently higher concentrations of circulating inflammatory markers [tumor necrosis factor-α, interleukin (IL)–6, IL-1ra, C-reactive protein, IL-8, chemokine ligand 3, and chemokine ligand 4].

**Conclusions:**

Collectively, these results highlight the central role of the gut in children with SAM. Malnutrition enteropathy may represent a suitable target for therapeutic interventions to improve clinical outcomes in this high-risk population.

## Introduction

Malnutrition is a major global health problem, implicated in almost half of deaths in children aged <5 y each year [1]. Severe acute malnutrition (SAM) is the most extreme form of undernutrition, affecting 19 million children each year, causing 400,000 deaths [1]. SAM is defined as a weight-for-height *z-*score (WHZ) <–3, mid-upper-arm circumference (MUAC) <115 mm, and/or bilateral nutritional edema, in a child aged between 6 and 59 mo [2]. Children are frequently treated in the community with ready-to-use therapeutic food, but if they have poor appetite, infections, or severe edema, they are categorized as having complicated SAM and require hospital management.

Children hospitalized with complicated SAM remain at particularly high risk of adverse outcomes during admission and after discharge. Across sub-Saharan Africa, reported mortality among children with complicated severe malnutrition remains as high as 10%–20% in hospitals and 10%–15% in the year after discharge [3,4]. In our Health Outcomes, Pathogenesis and Epidemiology of Severe Acute Malnutrition (HOPE-SAM) cohort in Zimbabwe and Zambia, 43.9% of children still met SAM criteria at hospital discharge, which was associated with >2-fold increased risk of death in the first year postdischarge [5]. Similar findings have been reported in Kenya, where 10.2% of HIV-negative children discharged after treatment for complicated SAM were readmitted with complicated SAM within 1 y [6]. These data highlight the ongoing vulnerability of children after hospitalization for SAM and the need to identify biological markers associated with relapse, readmission, and mortality.

Despite the large number of deaths and substantial morbidity associated with complicated SAM, little is known about the mechanisms that drive mortality in this high-risk group. Deaths are often ascribed to infectious causes [7]; however, SAM is a multisystem disease, with abnormalities in the gut, skin, endocrine, and immune systems [8]. The gut pathology, termed malnutrition enteropathy, is characterized by loss of villus height, crypt hyperplasia, and multiple epithelial derangements [9], leading to a leaky small intestine [10]. These histological features are not specific to malnutrition enteropathy and, therefore, overlap with other enteropathies, including environmental enteric dysfunction, HIV enteropathy, and persistent diarrhea [11]. Malnutrition enteropathy is distinguished primarily by its association with the anthropometric changes of SAM, which occurs in settings with high enteropathogen exposure and recurrent infection, rather than by pathognomonic small intestinal histology. The translocation of the cell-wall component LPS, otherwise known as endotoxin, and other pathogen-associated molecular patterns (PAMPs) is believed to drive innate immune activation [12]. It is hypothesized that this state of chronic inflammation reduces the ability of the immune system to respond to future infections through several processes leading to immunoparalysis, including LPS tolerance.

Systemic and intestinal inflammation are characteristic features of SAM. We have previously shown that children admitted to hospital with SAM display higher levels of circulating inflammatory biomarkers at admission compared with community controls, and that inflammation continues for 48 wk postdischarge despite nutritional recovery [13]. However, the role of the gut in this chronic inflammatory pathology needs to be further defined, as it is an attractive target for intervention using novel therapeutics [14]. Specifically, the relationship between intestinal permeability, bacterial translocation, and increased inflammation in SAM has not been assessed adequately to date.

In this substudy of a longitudinal multicenter observational study of children hospitalized with complicated SAM, we examine intestinal permeability and markers of intestinal translocation to assess the role of the gut in the pathogenesis of SAM. Intestinal permeability was assessed through a dual-sugar test to measure the urinary lactulose:mannitol ratio (LMR), a widely used noninvasive test of small intestinal barrier function, in which an increased LMR is indicative of increased intestinal permeability [15]. Circulating LPS was used as a marker of bacterial translocation, as LPS is a component of Gram-negative bacterial cell walls that may enter the circulation when gut barrier integrity is compromised. We also measured plasma biomarkers of systemic and vascular inflammation, as well as growth factors. Together, these analyses aim to define whether gut barrier dysfunction and intestinal translocation contribute to systemic inflammation and poor clinical outcomes in children with SAM.

## Methods

### Study design and participants

This was a substudy within the HOPE-SAM study, which has been described in detail elsewhere [16]. The HOPE-SAM protocol, standard operating procedures, and case report forms are available at https://osf.io/29uaw/. Briefly, HOPE-SAM was a longitudinal observational cohort study of children hospitalized for SAM at 3 tertiary referral hospitals in Zambia and Zimbabwe between August 2016 and March 2018. Eligible children were aged <60 mo, and admitted to a medical ward with complicated SAM, defined as WHZ <–3 and/or MUAC <115 mm and/or the presence of bilateral nutritional edema (in children aged >6 mo), according to WHO criteria. Children with malignancy, who died before study enrollment, or whose caregiver was not willing to learn the child’s HIV status, were not included in the study. This substudy enrolled children aged 6–59 mo, stratified by HIV status, to more intensive longitudinal specimen collection during follow-up; because the goal was to evaluate biomarkers of enteropathy, children with chronic gastrointestinal diseases were excluded.

To provide normative biomarker data for the local population, single samples of blood, stool, and urine (following LM administration) were collected from adequately nourished control children receiving inpatient or outpatient care at each site. Controls were aged 6–59 mo, with WHZ >–1, no acute illness or current infections, and known HIV status. Controls were broadly matched within age bands (6–11 mo, 12–23 mo, and 24–59 mo) and by HIV status to enrolled substudy children with SAM.

### Sample size

The sample size calculation for the main study is described elsewhere [16], and provided >80% power at 5% significance to detect absolute differences of 17% in binary outcomes, and 0.33 times the SD in continuous outcomes, assuming 15% mortality and 15% loss to follow-up.

### Laboratory analyses

#### LM measurements

A LM test was performed on cases and controls as previously described [17]. A solution of 250 mg/mL lactulose and 50 mg/mL mannitol was given orally at a dose of 2 mL/kg up to a maximum of 20 mL. Children were fasted for ≥30 min before and after ingestion of the LM solution, and all urine was collected for 2 h after administration of the solution. Chlorhexidine was added to the urine sample, which was kept in a cold box and transported to the laboratory for storage at –80°C with generator back-up. Urinary lactulose and mannitol concentrations were measured using liquid chromatography–MS/MS [TSQ Quantum Access; Thermo Scientific] at the University of East London, London, United Kingdom, with each sample having a double extraction, and mean values were used. Results for lactulose or mannitol that were below the limit of quantification were included at the level of the lowest detectable level divided by √2, as described previously for out-of-range results [18], if there was a valid result for the other sugar.

#### LMR calculation

A LMR was calculated using the posttest urinary concentration of the sugars, based on the equation below, the derivation of which is outlined in Supplementary Equation 1.

$$lactulos\mathrm{e:}\;mannitol\;ratio=\frac{{lactulose}_{conc.\;urine}}{{5\times mannitol}_{conc.\;urine}}$$

#### LPS measurement

LPS was measured in plasma, after blood collection into endotoxin-free EDTA tubes. All samples were measured in 1 laboratory in Zimbabwe. This used the limulus amoebocyte lysate (LAL) assay (Associates of Cape Cod Inc.), based on clotting components harvested from the blood of horseshoe crabs [19]. Plasma was centrifuged at 15,000 × *g* for 5 min at room temperature to sediment cells/particles in the samples. Cell-free plasma was diluted 1:10 with endotoxin-free LAL reagent water. Diluted samples were heated to 70°C for 30 min to denature any proteins in the plasma, which may affect the LAL assay. A total of 50 μL of diluted sample was analyzed in duplicate, following manufacturer instructions using Glucashield buffer, with results read using a kinetic plate reader at 410 nm with a read every 50 s for 90 min.

#### Plasma ELISAs and Luminex assay

Plasma ELISA and Luminex assays were carried out as previously described and reported [13]. ELISAs were undertaken according to the manufacturer’s specification for the following analytes: C-reactive protein and sCD14 following 1:200 dilution, and sCD163 following 1:40 dilution (R&D Quantikine); LPS-binding protein (LBP) following 1:800 dilution (R&D Duoset); intestinal fatty acid–binding protein following 1:5 dilution (Hycult Biotech); and glucagon-like peptide–2 (GLP-2) at 1:1 dilution (Merck Millipore).

For Luminex assays, all samples were analyzed in Zimbabwe using 1 custom 25-plex Luminex panel (R&D Systems), with full results reported elsewhere [13]. The Luminex panel and codes are shown in Supplemental Table 1. Plasma was analyzed in technical duplicates on a Luminex Magpix machine (Luminex Corp). Results above the limit of detection were repeated at a lower concentration, and results below the limit of detection at the highest concentration (1:2) were included in analyses using a value calculated from the lowest limit of detection for that analyte divided by √2.

### Statistics

#### Longitudinal analysis

Longitudinal LPS and LMR data from the 4 time points between baseline and 48 wk were analyzed using mixed effects modeling using maximum likelihood estimation of coefficients, with each logged result along with: HIV, baseline edema, any diarrhea (yes/no), WHZ score, country, baseline age, and whether the child was breastfeeding. These covariates were offered to the model as fixed effects, and the participant identifier as the random effect. These covariates were used in previous analyses, which were identified using directed acyclic graph modeling [13]; we excluded pneumonia and sepsis variables from the current analysis, given the small numbers in these groups. The longitudinal values from children with SAM were compared with the control values of LPS and LMR from 1 time point.

#### Relationship of LPS and LMR with other biomarkers

The impact of higher LPS level was assessed by regressing the biomarker under investigation with the log_10_-transformed LPS level. To account for confounding effects, the same group of covariates was also offered to the model, as for the longitudinal analysis.

Biomarkers were normalized by log_10_ transformation of the raw biomarker concentration. For the majority of variables, this was the most appropriate transformation from the ladder of powers assessment. The reported *P* value was adjusted using the Benjamini–Hochberg false discovery rate correction. Any difference was considered significant if the adjusted *P* value was <0.05.

#### Survival analysis

A hazard ratio was calculated using Cox’s proportional hazards model, and the proportional hazards assumption was verified using Schoenfeld residuals.

#### Missing data

Missing variable data for LMR results were predominantly related to the technical difficulty of the test, requiring urine collection for ≥2 h after ingestion of a LM solution. LPS testing was not conducted when there was insufficient remaining, as this was one of the last tests to be run on stored samples. To explore the impact of any departure from a missing at random assumption, a sensitivity analysis was carried out, with missing variables imputed using the 10th and 90th percentiles of the observed variable at each time point, and the effect of these extreme values on overall conclusions was explored. Children who had died did not have any further data imputed.

All analyses were carried out using Stata (version 19.5).

#### Ethics

Ethical approval for the study was obtained from the University of Zambia Biomedical Research Ethics Committee (010-02-16), and the Medical Research Council of Zimbabwe (MRCZ/A/2044). The ethics committee of Queen Mary University of London provided an advisory review. Written informed consent from caregivers was obtained in local languages.

#### Role of funders

The funding bodies had no role in the study design, implementation, or analysis and interpretation of the data.

## Results

**FIGURE 1:**
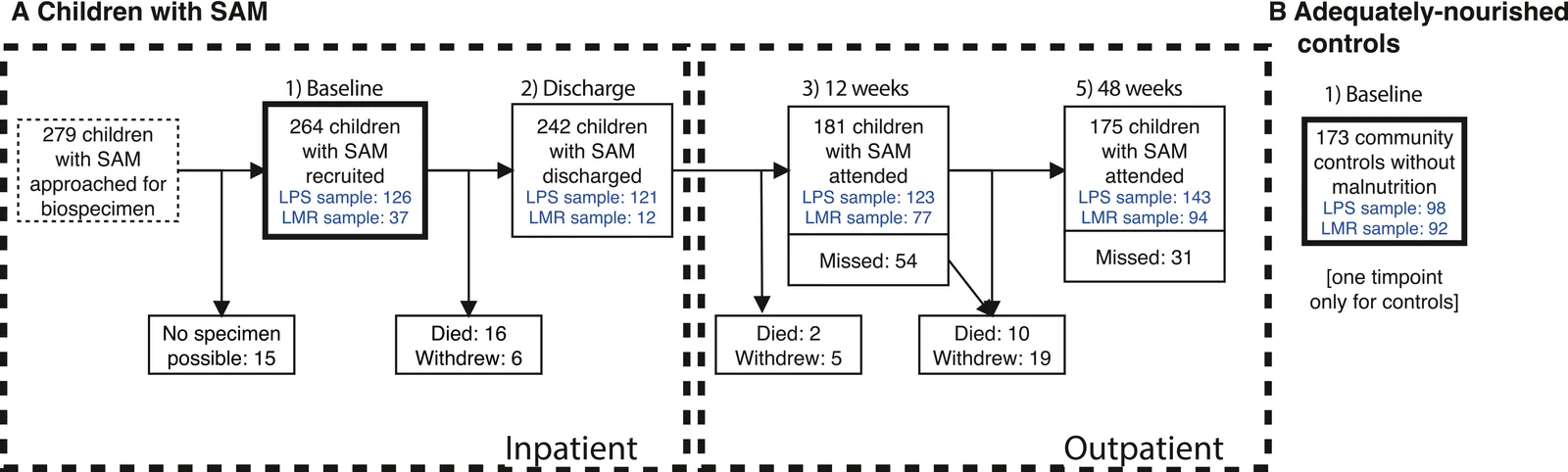
Flow of participants with LPS and LMR samples in the HOPE-SAM study. The flow diagram progresses the 264 patients recruited in the HOPE-SAM study. Children with SAM were enrolled during hospitalization and followed at discharge, then at 2, 4, 12, 24, and 48 wk, with samples collected at 12 and 48 wk. Blood was used to measure circulating LPS, and urine was collected after oral ingestion of a dual sugar solution to measure the urinary LMR. The number of children with successful specimen collection at each time-point is shown in blue. LMR, lactulose:mannitol ratio; HOPE-SAM, Health Outcomes, Pathogenesis and Epidemiology of Severe Acute Malnutrition.

A total of 437 children were enrolled in the enteropathy substudy of the HOPE-SAM study in Zimbabwe and Zambia, as shown in Figure 1. Of these, 264 children hospitalized with SAM were followed longitudinally, whereas 173 adequately nourished community control children underwent a single cross-sectional evaluation. Demographics of children with available samples, who were included in this analysis, are shown in Table 1, fully expanded in Supplemental Table 2. Children with available samples, compared with the overall substudy population, were more likely to be from Zimbabwe, spent fewer days in hospital, and had lower mortality (Table 1). Differences between the enteropathy substudy population and the overall HOPE-SAM cohort have already been published elsewhere [13].

**TABLE 1:** Characteristics of participants

	A) Children with complicated SAM					B) Adequately-nourished controls				
	Overall cohort	LPS sample available^1^	*P* value	LMR sample available^1^	*P* value	Overall cohort	LPS sample available	*P* value	LMR sample available	*P* value
Number of participants, *n* (%)	264	202 (76.5)		132 (50.0)		173	98 (56.6)		93 (53.8)	
Site: Zimbabwe, *n* (%)	198 (75.0)	180 (89.1)	<0.001	123 (93.2)	<0.001	103 (59.5)	98 (100.0)	<0.001	85 (91.4)	<0.001
Base age (mo), median (IQR)	18.4 (13.2, 22.8)	19.2 (13.4, 23.6)	0.027	20.1 (14.9, 24.9)	0.004	28.0 (18.0, 42.5)	30.0 (18.0, 44.0)	0.272	30.0 (18.5, 46.0)	0.149
Male, *n* (%)	141 (53.4)	109 (54.0)	0.746	78 (59.1)	0.064	100 (57.8)	57 (58.2)	0.913	57 (61.3)	0.317
HIV positive, n (%)	60 (22.7)	45 (22.3)	0.753	29 (22.0)	0.769	60 (34.7)	31 (31.6)	0.335	27 (29.0)	0.092
WHZ at baseline, median (IQR)	–3.0 (–4.0, –1.6)	–3.0 (–4.0, –1.4)	0.729	–2.8 (–3.9, –1.3)	0.116	0.2 (–0.3, 0.9)	0.2 (–0.2, 0.8)	0.336	0.2 (–0.2, 1.0)	0.304
Edema present, *n* (%)	177 (67.3)	138 (68.3)	0.523	88 (66.7)	0.826	—	—	—	—	—
Days in hospital [median (IQR)]	11.0 (8.0, 16.0)	10.0 (7.0, 14.0)	<0.001	10.0 (7.0, 14.0)	0.017	—	—	—	—	—
Diarrhea present, *n* (%)	129 (48.9)	100 (49.5)	0.707	67 (50.8)	0.538	—	—	—	—	—
Pneumonia present, *n* (%)	22 (9.2)	14 (7.7)	0.161	8 (6.7)	0.193	—	—	—	—	—
Sepsis present, *n* (%)	11 (4.6)	8 (4.4)	0.805	5 (4.2)	0.779	—	—	—	—	—
Died, *n* (%)	28 (11.2)	10 (5.0)	<0.001	2 (1.5)	<0.001	—	—	—	—	—
Poor outcome > 48 wk^2^, *n* (%)	45 (18.7)	32 (15.8)	0.010	19 (14.4)	0.061	—	—	—	—	—
LPS/LMR result also present, n (%)	—	127 (62.9)	<0.001	127 (96.2)	<0.001	—	81 (82.7)		81 (87.1)	
Number of time points of LMR/LPS per patient (mean)		2.54		1.67			1.00		1.00	
Total samples (*n*)		513		221			98		93	

Characteristics of children with severe acute malnutrition (SAM) (A) and adequately nourished controls (B) are shown, comparing those who had available samples for the current analysis, with the whole enrolled population. Statistical differences between the groups with and without a sample are shown by the *P* value. Categorical variables were tested using χ^2^ testing. Continuous variables were tested using regression. Results were considered significant and bolded if the *P* value was <0.05.

Abbreviations: LMR, lactulose:mannitol ratio; SAM, severe acute malnutrition; WHZ, weight for height *z*-score.

Full details of those children without samples are included in Supplemental Table 2.

1 Sample present at any time point.

2 Poor outcome defined as death or readmission at any point during 48 wk postdischarge.

### LMRs are elevated in children with complicated SAM

**FIGURE 2:**
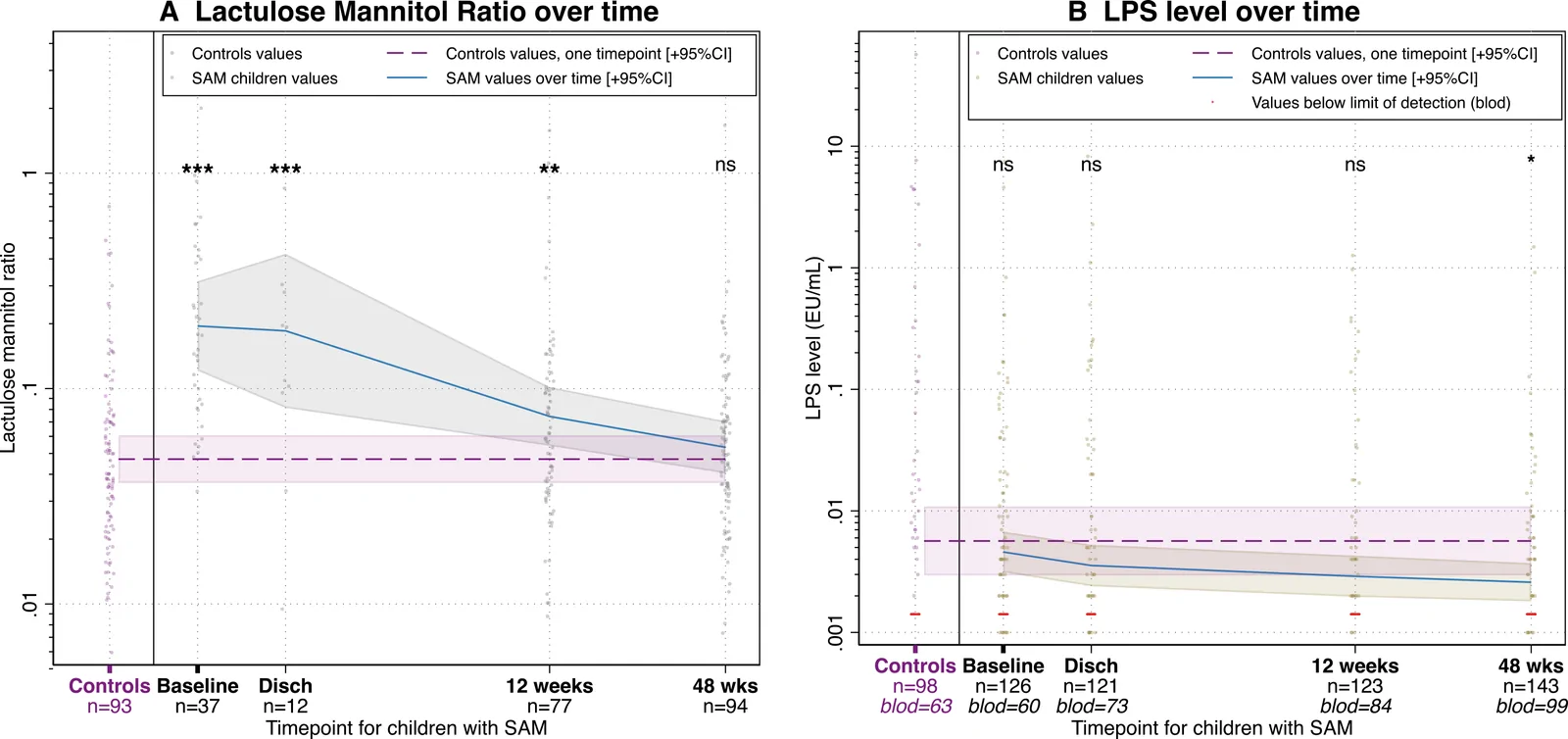
Changes over 48 wk in children with SAM compared with normative values from adequately nourished controls for (A**) lactulose mannitol ratio** (LMR) and (B) LPS plasma level. The LPS values which are below limit of detection (blod) are included in the analysis, by assigning a value calculated as (limit of detection divided by √2). Results are overlaid with 95% CIs based on longitudinal mixed effects modeling. Statistical significance shown based on the Mann–Whitney *U* test at each time point. ∗*P* < 0.05; ∗∗*P* < 0.01; ∗∗∗*P* < 0.001; Results were not adjusted for any covariates; adjusted results are similar and shown in Supplemental Figure 1. CI, confidence interval; ns, not significant; SAM, severe acute malnutrition.

Lactulose and mannitol urinary concentration results were available for 132 of 264 (50%) children with SAM, totaling 221 samples, averaging 1.7 time points per child (Table 2). Six lactulose results and 1 mannitol result included in the analysis were below the limit of detection. LMR results were also available for 92 of 172 (53%) controls. Children with SAM had significantly higher concentrations of urinary lactulose at baseline and discharge, whereas concentrations of mannitol were nonsignificantly lower compared with those of adequately nourished controls. LMR values were higher in children with SAM at baseline, discharge, and 12 wk, suggesting increased intestinal permeability; values normalized to the level of the adequately nourished controls between 12 and 48 wk (Figure 2). In longitudinal modeling, >48 wk, children with SAM had a log_10_ LMR of +0.39, which was 2.5 times higher [95% confidence interval (CI): 1.4, 4.5] than the LMR of control children.

**TABLE 2:** Results of urinary concentrations of lactulose, mannitol, and the lactulose:mannitol ratio, and plasma LPS levels

		Urinary lactulose (μg/mL)	Urinary mannitol (μg/mL)	Lactulose:Mannitol ratio	Plasma LPS level (EU/mL)
A. All time points	Controls				
	Obs	*n* = 93	*n* = 93	*n* = 93	*n* = 98
	Median result	70	383	0.040	0.0014
	IQR	(30, 132)	(144, 695)	(0.024, 0.074)	(0.0014, 0.009)
	SAM children				
	Obs	*n* = 220	*n* = 220	*n* = 220	*n* = 513
	Median	108	297	0.069	0.0014
	IQR	(38, 244)	(110, 650)	(0.036, 0.14)	(0.0014, 0.004)
B. Each time point individually	Baseline: SAM children				
	Number of samples	*n* = 37	*n* = 37	*n* = 37	*n* = 126
	Median result	244	207	0.18	0.0014
	IQR	(108, 373)	(135, 568)	(0.085, 0.41)	(0.0014, 0.009)
	Significance over controls^1^	<0.001	0.273	<0.001	0.743
	Discharge: SAM children				
	Number of samples	*n* = 12	*n* = 12	*n* = 12	*n* = 121
	Median result	188	123	0.19	0.0014
	IQR	(105, 314)	(96, 428)	(0.099, 0.58)	(0.0014, 0.004)
	Significance over controls^1^	0.003	0.089	<0.001	0.259
	12 wk: SAM children				
	Number of samples	*n* = 77	*n* = 77	*n* = 77	*n* = 123
	Median result	94	294	0.062	0.0014
	IQR	(23, 192)	(58, 539)	(0.034, 0.12)	(0.0014, 0.002)
	Significance over controls^1^	0.790	0.111	0.007	0.074
	48 wk: SAM children				
	Number of samples	*n* = 94	*n* = 94	*n* = 94	*n* = 143
	Median result	88	352	0.049	0.0014
	IQR	(32, 206)	(168, 753)	(0.032, 0.083)	(0.0014, 0.002)
	Significance over controls^1^	0.37	0.946	0.308	0.013

Number of samples, results, and IQR are shown for SAM children and controls at (A) all time points, and (B) at each time point individually for the children with SAM. Results for the LPS include the results below the limit of detection.

Abbreviation: SAM, severe acute malnutrition.

1 Significance is shown as the *P* value for the rank-sum test on untransformed data.

### Blood LPS concentrations are similar in SAM and controls

There were longitudinal plasma samples available for analysis of circulating LPS in 209 children with SAM, totaling 649 samples, with an mean of 3.1 time points per child (Table 2). Cross-sectional LPS measurements were available in 98 of 173 (57%) controls. There was no evidence that LPS concentrations differed between children with SAM and adequately nourished controls, with median values across all groups of 0.0014 EU/mL with slightly different IQRs as per Table 2, as shown in Figure 2A. There were a notable number of LPS results below the limit of assay detection (379 of 611, 62%), but inferences were similar when these results were excluded from analyses (Supplemental Figure 2).

### High circulating LPS is associated with higher levels of acute inflammatory cytokines

**FIGURE 3:**
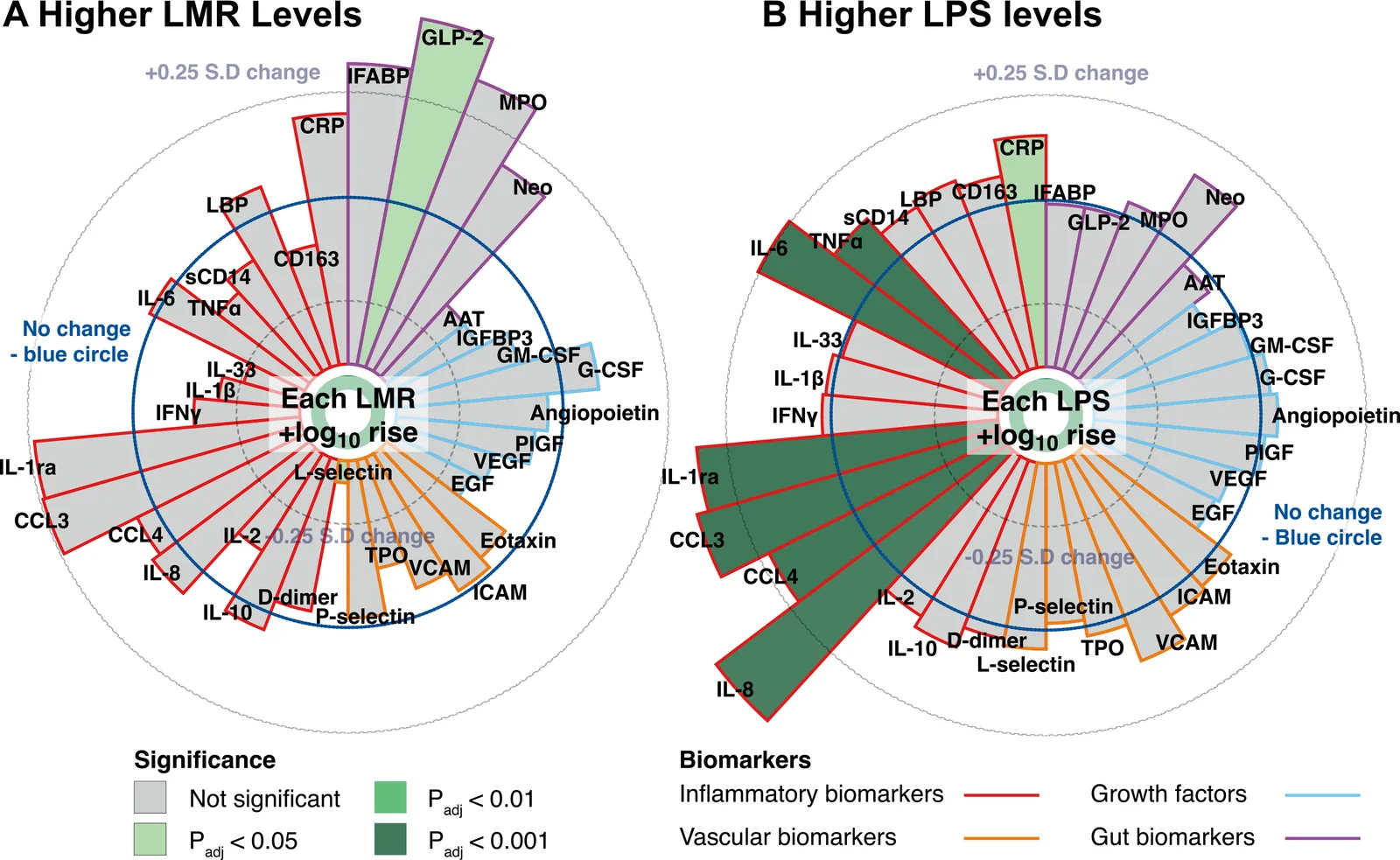
Differences in biomarker concentrations in children with SAM with (A) each log_10_ LMR increase (*n* = 220), and (B) with each log_10_ LPS concentration increase (*n* = 513). Variables adjusted for: HIV, edema, diarrhea, weight-for-height *z*-score, country, age (mo), and breastfeeding status. Adjusted *P* value derived from Benjamini–Hochberg false discovery rate correction. SD of the log_10_-transformed biomarker. Full numerical results, as well as exact numbers for each biomarker, are shown in Supplemental Tables 4 and 5. CCL3, chemokine ligand 3; CCL4, chemokine ligand 4; CCL11, chemokine ligand 11; G-CSF, granulocyte colony-stimulating factor; GLP-2, glucagon-like peptide–2; GM-CSF, granulocyte-macrophage colony-stimulating factor; ICAM, intercellular adhesion molecule; IFABP/I-FABP, intestinal fatty acid–binding protein; IFNγ, interferon gamma; IGFBP3, insulin-like growth factor binding protein 3; LBP, LPS-binding protein; LMR, lactulose:mannitol ratio; MPO, myeloperoxidase; PlGF, placental growth factor; SAM, severe acute malnutrition; TPO, thrombopoietin; VCAM, vascular cell adhesion molecule; VEGF, vascular endothelial growth factor.

The association between LPS concentrations in children with SAM and a panel of 35 other biomarkers (Supplemental Table 1) was next explored. After correction for multiple hypothesis testing and adjustment for confounders (HIV, edema, diarrhea, WHZ, country, age in months, and breastfeeding status), across all time points, for each log_10_ rise in circulating LPS, there was a significant increase in TNFα, IL-6, IL-1ra, IL-8, chemokine ligand 3 (CCL3), and chemokine ligand 4 (CCL4), as shown in Figure 3, with full results in Supplemental Table 4. This supports the hypothesis that LPS drives increased innate immune responses in children with SAM.

### High LMR is associated with higher levels of intestinal markers

We next explored the relationship between LMR, which reflects gut permeability, and plasma biomarkers. After correction for multiple hypothesis testing and adjustment for confounders, each log_10_ rise in LMR was associated with a significant increase in GLP-2, which is associated with re-epithelialization and gastric motility, and a decrease in L-selectin, as shown in Figure 3, with full results in Supplemental Table 4. However, there was no relationship between LPS concentrations and LMR, regardless of whether LPS levels below the limit of detection were included or excluded (Supplemental Figure 3).

### Higher LMR is associated with poor clinical outcomes

We next used Cox proportional hazards models to evaluate whether LMR or circulating LPS concentrations are associated with poor clinical outcomes, defined as death or readmission at any point to week 48. LMR was associated with poorer clinical outcomes, with each log_10_ rise in inpatient LMR associated with 6.1 (95% CI: 1.03, 36.8) times higher hazard of death or readmission over the following 48 wk (Table 3). Because of the small number of events, an adjusted analysis was not carried out. Results were similar when restricted to baseline values of LMR/LPS (Supplemental Table 6). There was no relationship between LPS concentration during hospitalization and subsequent clinical outcomes.

**TABLE 3:** Hazard ratios based on hospital levels of LMR and LPS

	Unadjusted			Numbers: Failures/total *n*
	Hazard Ratio	95% CI	*P* value	
Inpatient LMR (log_10_)	6.14	(1.03,36.8)	0.047	5/45
Inpatient LPS (EU/mL log_10_)	0.88	(0.40, 1.88)	0.718	27/149

Hazard ratios for a composite poor outcome of death or readmission through 48 wk postdischarge. The first measurement available during the hospital admission was used (i.e., a discharge value was used if a baseline level was not available).

Abbreviations: CI, confidence interval; LMR, lactulose mannitol ratio.

## Discussion

In this prospective observational substudy of children admitted with SAM in 3 southern African hospitals, we examined relationships between the gut and systemic inflammation by measuring intestinal permeability, circulating LPS concentrations, and a panel of inflammatory biomarkers. Our results suggest that gut pathology is implicated in SAM, consistent with biopsy studies, which confirm that malnutrition enteropathy is almost universal in this population of children [10,20]. We had 3 main findings. First, intestinal permeability, measured by the urinary LMR, was raised compared with adequately nourished controls at baseline, and only resolved slowly over the following 48 wk postdischarge. Second, higher gut permeability was associated with an increased risk of death or readmission over the following year. Third, higher concentrations of LPS were independently associated with higher circulating inflammatory markers, including TNF-α, C-reactive protein (CRP), L-6, IL-8, CCL3, and CCL4. Taken together, these findings suggest a complex pathological pathway linking gut pathology and systemic inflammation, which persists after hospital discharge, and may contribute to poor long-term outcomes in children with complicated SAM.

Intestinal permeability can be noninvasively measured using a combination of 2 sugars, a larger nonabsorbable sugar such as lactulose, and a smaller absorbable sugar such as mannitol or rhamnose. This functional test has been conducted in multiple populations in low- and middle- income countries (LMICs), with LMR generally higher than in children from Western countries [15]. Permeability has been noted to be increased in children with severe malnutrition since the 1960s [[21], [22], [23]], with some studies showing an inverse relationship with anthropometry markers such as weight-for-age and height-for-age *z*-scores [15], but many studies have been limited by a lack of suitable community controls. The mechanism underlying increased intestinal permeability is unclear, but breaches in the gut epithelium have been described in malnutrition enteropathy [10]. There also may be contributions from gut-resident and infiltrating immune cells, and the gut-associated lymphoid tissue, which can release proinflammatory cytokines; the intestinal microbiota, which can help drive a proinflammatory response; and/or the enteric nervous system [24]. Although children with SAM have clear evidence of malnutrition enteropathy, this is histologically indistinguishable except in severity from environmental enteropathy, which is almost universal in community children [25]. Many children with SAM also present with diarrhea (48% in this cohort), which is associated with intestinal permeability [26], although it is unclear whether this is a cause or a consequence of the changes, as diarrhea in children with SAM may be caused by organisms that are not typically considered pathogenic [[27], [28], [29]], or lack an obviously identifiable pathogen [27]. A combination of inflammatory and infective processes is likely to drive a cycle of malnutrition enteropathy, which prevents further epithelial healing [30].

The consequences of increased permeability include loss of barrier function of the gut. This can lead to increased translocation of intestinal products (including LPS and other PAMPs, as well as viable bacteria), and reduced functional absorption of nutrients, with villous height inversely related to permeability biomarkers in Zambian children [31]. Intestinal permeability has been associated with poorer nutritional outcomes in children with SAM [21], although it has been suggested that increased permeability may have more impact in specific populations such as children living with HIV [32]. Increased permeability has also been associated with decreased linear growth velocity of children with complicated SAM after discharge, either due to decreased absorption or the deleterious effects of increased inflammation. Children with increased intestinal permeability had significantly higher circulating GLP-2, a protein secreted by the terminal ileum, which preserves mucosal integrity and promotes growth and repair of the intestine [33], suggesting an attempt to remediate this process.

LPS, otherwise known as endotoxin, is a component of the Gram-negative bacterial cell wall and is highly immunogenic. It has been detected in plasma during Gram-negative infections [34], as well as trauma [35], obesity [36], and even cardiac arrest [37], so its presence is not necessarily indicative of infection. Plasma LPS in noninfective cases is plausibly attributed to translocation of bacterial products across the gut wall, whose permeability is increased in critical illnesses. Exposure to LPS can lead to strong innate immune responses [38], but repeated exposure can lead to reduced responses through a process called LPS tolerance that is likely epigenetically driven [39,40]. This might contribute to the higher levels of circulating inflammatory biomarkers seen in children with SAM [13], as well as the increased susceptibility to infectious mortality, which remains high for ≥1 y after discharge [41]. Although we found expected increases in plasma inflammatory markers in those with more circulating LPS (Supplemental Table 4), we did not demonstrate increased LPS levels in children with SAM (Supplemental Table 3). This may represent a genuine lack of difference in children with SAM, or may reflect the fact that LPS, which is highly immunogenic, is swiftly bound and inactivated by proteins such as LBP, antibodies, or other substances such as some lipids [42]. We have previously shown heightened bacterial binding by innate immune cells in vitro, suggesting that circulating cells may be better at “mopping up” circulating LPS while also producing lower levels of proinflammatory cytokines in children with SAM compared with controls [43]. This could plausibly contribute to the uncoupling of LPS and its relationship with biomarker concentrations. Furthermore, the acute level of LPS level measured may not reflect chronic endotoxemia to which the child may have been exposed.

Similarly, there was no relationship between LMR and circulating LPS levels in this study. Despite the theoretical mechanistic relationship, this lack of correlation has been observed in other studies in similar but not identical populations [44], whereas some other studies have reported elevated Endotoxin-Core Antibody (EndoCAb) IgG, a marker of LPS exposure, correlating with intestinal permeability [45]. In a systematic review assessing the relationship between the hypothesized domains of environmental enteropathy, there was little direct evidence to support the pathway from intestinal permeability to microbial translocation [12].

LM testing is notoriously difficult to undertake and to compare across studies due to procedural differences, and the LMR is affected by multiple factors, including age, intestinal motility, gastric emptying times, and renal clearance [15]. Furthermore, lactulose permeability may not indicate the ability of bacteria or microbial products to translocate, due to differences in the molecular weight of lactulose (324 Da) compared with LPS (10–20 kDa) [46]. Although LMRs are higher in children with SAM, translocated LPS may be neutralized by LBP (CD14), EndoCAb, and by antimicrobial peptides [47], and/or endocytosed by immune cells. Neutralization may help explain why many LPS values were below the threshold of detection, and may limit the utility of measuring LPS directly rather than using markers of longer-term exposure to LPS. The relationship between gut inflammation and systemic inflammation may also be driven by factors other than the translocation of bacterial products. Changes in the gut microbiota, along with altered metabolic byproducts, direct effects of cytokines produced by intestinal lymphoid tissue, as well as indirect effects of other products such as bile acids, short-chain fatty acids, and mucus alterations, could all contribute to the relationship between increased intestinal inflammation and systemic inflammatory sequelae [48].

Our findings suggest biomarkers of gut permeability measured by LMR may have prognostic utility in this hospitalized SAM population, as higher inpatient LMR was associated with increased risk of death or hospital readmission. Although our measurements required MS, novel noninvasive techniques such as transcutaneous fluorescence spectroscopy [49] may permit bedside assessment of gut permeability, potentially improving feasibility in low-resource settings. This raises the possibility that such biomarkers could be used to identify higher-risk children who may benefit from more intensive monitoring or prolonged inpatient care, and could ultimately inform more individualized approaches to SAM management. Treatment of enteropathy remains an underexplored area: the relationship between gut and systemic inflammation has been targeted in novel trials of mesalazine [50], and more recently in a trial of teduglutide, budesonide, n-acetylglucosamine, and colostrum, in children with complicated SAM [20]. Although some interventions reduced systemic inflammation following the gut-focused interventions [51], these studies were not powered to show if biomarker changes translated into improved clinical outcomes.

Limitations of this study include missing data related to the technical requirements of the tests deployed. The LM test requires ingestion of a sugar solution, as well as collection of urine for 2 h after administration, in young, sick children. Administration of LM solution to children with suppressed appetite, as well as practical difficulties in collecting urine from small children, led to missing LMR in half the cohort, particularly in girls and younger children (Table 1). Our sensitivity analysis confirmed that inferences from the longitudinal results were unaltered (Supplemental Table 7), but the survival analysis could be affected if the data were not missing at random, and inferences about the generalizability of the results should, therefore, be guarded. We did not test for specific enteric pathogens in these children, and permeability and/or outcomes may be influenced by specific pathogens [52]. Other limitations include the timing of recruitment: many caregivers required 24–48 h to consider enrollment in the study, and LMR urinary testing was only carried out on weekdays (with full timing of LMR tests shown in Supplemental Figure 4), so this cohort is not reflective of children who have immediately been admitted with SAM, in whom gut biomarkers may differ.

In conclusion, this study showed that higher intestinal permeability in SAM compared with controls persists for ≤1 y, and is associated with poorer clinical outcomes, and increased biomarkers of intestinal repair. LPS concentrations were not acutely correlated with intestinal permeability, but were associated with systemic inflammation. Collectively, these results suggest that the gut has an important role in the pathophysiology of SAM. Novel interventions targeting the gut, therefore, have biological plausibility and should be tested in the future for effects on clinical outcomes, including mortality, readmission to hospital, and nutritional recovery.

## Author contributions

The authors’ responsibilities were as follows – MB-D, BA, KJN, PK, AJP: designed and oversaw the study; MB-D, BA, CDB, JPS, KJN, PK, AJP: contributed to funding; MB-D BA, DN, FDM: collected data; JPS, JT, CD, KM, EB, RN, TH, BC, AJP: contributed to analysis; and all authors: critically reviewed and approved the manuscript.

## Declaration of generative AI and AI-assisted technologies in the writing process

The authors declare that no generative AI or AI-assisted technologies were used in the writing of this manuscript.

## Data availability

Source data have been deposited in Figshare under accession code: 10.6084/m9.figshare.29263562 https://figshare.com/s/ae8bc49d50252bcf29f1

## Funding

This work was supported by Medical Research Council (grant MR/K012711/1) to AJP, Wellcome (107634/Z/15/Z) to MBD, Wellcome (108065/Z/15/Z) to AJP, Wellcome (220566/Z/20/Z) to JPS, A joint award from Wellcome and the Royal Society (206225/Z/17/Z) to CDB, National Institute of Health and Care Research (NIHR201813) to MBD and AJP, and UNICEF Zimbabwe (ZIM/PCA201721/PD2019158) to AJP.

## Conflict of interest

The authors report no conflicts of interest.
