# Supplementary material for: Intestinal Permeability Is Associated with Mortality and Readmission in Children Hospitalized with Severe Acute Malnutrition
Source: J Nutr. 2026 Jun 6;156(8):101655. doi: 10.1016/j.tjnut.2026.101655 (PMC13494158; doi:10.1016/j.tjnut.2026.101655)
Supplement: Supplementary file 1 [file mmc1.docx]

**Supplementary Material**

**Intestinal permeability is associated with mortality and readmission in children hospitalised with severe acute malnutrition**

**Sturgeon et al.**

**Supplementary Material: Supplementary Figures/Tables**

[Supplementary Table 1: List of the biomarkers analysed	2](#_Toc228973539)

[Supplementary Table 2: Full details of children with and without samples	3](#_Toc228973540)

[Supplementary Table 3: Longitudinal modelling of LPS and LMR results showing changes over the controls	4](#_Toc228973541)

[Supplementary Table 4: Changes in biomarker level for each log_10_ increase in plasma LPS concentration	5](#_Toc228973542)

[Supplementary Table 5: Changes in biomarker level for each log_10_ increase in lactulose:mannitol ratio	6](#_Toc228973543)

[Supplementary Table 6: Results of effect of baseline levels of LMR and LPS on death or hospital readmission	7](#_Toc228973544)

[Supplementary Table 7: Sensitivity analysis examining the potential impact of missing data if there were a departure from the missing at random assumption	8](#_Toc228973545)

[Supplementary Table 8: Derivation of the lactulose:mannitol ratio equation	9](#_Toc228973546)

[Supplementary Figure 1: Adjusted results for changes over time in children with SAM compared with adequately nourished controls for A) LPS level and B) LMR	10](#_Toc228973547)

[Supplementary Figure 2: Results for LPS analysis, with the out-of-range (OOR) results excluded, both A) unadjusted, and B) adjusted for variables shown	11](#_Toc228973548)

[Supplementary Figure 3: Graphs showing the correlation between a) LMR and LPS level, b) Urinary lactulose concentration and LPS level, and c) urinary mannitol concentration and LPS levels	12](#_Toc228973549)

[Supplementary Figure 4: Timing of LM urinary testing compared with baseline HOPE-SAM visit	13](#_Toc228973550)

| **Short name** | **Full name** | **Method** |
| --- | --- | --- |
| **CRP** | **C-reactive protein** | **ELISA** |
| **sCD14** | **Soluble CD14** | **ELISA** |
| **LBP** | **Lipopolysaccharide-binding-protein** | **ELISA** |
| **sCD163** | **Soluble CD163** | **ELISA** |
| **TNFα** | **Tumour necrosis factor-alpha** | **Luminex panel** |
| **IL-6** | **Interleukin-6** | **Luminex panel** |
| **IL-33** | **Interleukin-33** | **Luminex panel** |
| **IL-8** | **Interleukin-8** | **Luminex panel** |
| **L-selectin** | **L-selectin** | **Luminex panel** |
| **IL-10** | **Interleukin-10** | **Luminex panel** |
| **EGF** | **Epidermal growth factor** | **Luminex panel** |
| **VEGF** | **Vascular endothelial growth factor** | **Luminex panel** |
| **IL-2** | **Interleukin-2** | **Luminex panel** |
| **IL-1β** | **Interleukin-1β** | **Luminex panel** |
| **IFN-γ** | **Interferon-gamma** | **Luminex panel** |
| **IL-1ra** | **Interleukin -1 receptor antagonist** | **Luminex panel** |
| **CCL3** | **Chemokine (C-C motif) ligand 3** | **Luminex panel** |
| **CCL4** | **Chemokine (C-C motif) ligand 4** | **Luminex panel** |
| **D-dimer** | **D-dimer** | **Luminex panel** |
| **GM-CSF** | **Granulocyte-macrophage colony-stimulating factor** | **Luminex panel** |
| **P-selectin** | **P-selectin** | **Luminex panel** |
| **GCSF** | **Granulocyte colony-stimulating factor** | **Luminex panel** |
| **TPO** | **Thrombopoietin** | **Luminex panel** |
| **VCAM-1** | **Vascular cell adhesion molecule 1** | **Luminex panel** |
| **ICAM-1** | **Intercellular adhesion molecule 1** | **Luminex panel** |
| **Angiopoietin** | **Angiopoietin-1** | **Luminex panel** |
| **PlGF** | **Placental growth factor** | **Luminex panel** |
| **Eotaxin** | **Eotaxin-1 (CCL11)** | **Luminex panel** |
| **IGFBP-3** | **IGF-binding protein 3** | **Luminex panel** |

Supplementary Table 1: List of the biomarkers analysed

The first Luminex panel was one single 25-plex panel (Assay code: UgRYKY2M; R&D Systems, MN, USA). This was used to analyse the week 48 samples of children in an enteropathy sub-study section (n=156) and all healthy controls (n=157)

The same Luminex assay was no longer available from the manufacturer, so a 24-plex panel without P-selectin was run for the remaining week 48 samples (n=170), Assay code: B829YuzuZ. The same ELISAs were done on all samples.

|  | **Children with SAM** | | | | | | | **Adequately nourished controls** | | | | | | |
| --- | --- | --- | --- | --- | --- | --- | --- | --- | --- | --- | --- | --- | --- | --- |
|  | **Overall cohort** | **LPS sample available*** | **LPS sample unavailable** | **P-value** | **LMR sample available*** | **LMR sample unavailable** | **P-value** | **Overall cohort** | **LPS sample available*** | **LPS sample unavailable** | **P-value** | **LMR sample available** | **LMR sample unavailable** | **P-value** |
| Number of participants | 264 | 202 (76.5%) | 62 (23.5%) |  | 132 (50.0%) | 132 (50.0%) |  | **173** | 98 (56.6%) | 75 (43.4%) |  | 93 (53.8%) | 80 (46.2%) |  |
| Site: Zimbabwe | 198 | **180 (89.1%)** | **18 (29.0%)** | **<0.001** | **123 (93.2%)** | **75 (56.8%)** | **<0.001** | **103 (59.5%)** | **98 (100.0%)** | 5 (6.7%) | **<0.001** | **85 (91.4%)** | 18 (22.5%) | **<0.001** |
| Base age (median [IQR], months) | 18.4 | **19.2 [13.4,23.6]** | **17.4 [12.2,20.3]** | **0.027** | **20.1 [14.9,24.9]** | **17.4 [12.4,21.5]** | **0.004** | **28.0 [18.0,42.5]** | 30.0 [18.0,44.0] | 26.3 [18.0,41.2] | 0.272 | 30.0 [18.5,46.0] | 24.5 [17.5,41.0] | 0.149 |
| Male | 141 | 109 (54.0%) | 32 (51.6%) | 0.746 | 78 (59.1%) | 63 (47.7%) | 0.064 | **100 (57.8%)** | 57 (58.2%) | 43 (57.3%) | 0.913 | 57 (61.3%) | 43 (53.8%) | 0.317 |
| HIV positive | 60 (22.7%) | 45 (22.3%) | 15 (24.2%) | 0.753 | 29 (22.0%) | 31 (23.5%) | 0.769 | **60 (34.7%)** | 31 (31.6%) | 29 (38.7%) | 0.335 | 27 (29.0%) | 33 (41.2%) | 0.092 |
| WHZ at baseline (median [IQR]) | -3.0 [-4.0,-1.6] | -3.0 [-4.0,-1.4] | -2.9 [-4.0,-2.0] | 0.729 | -2.8 [-3.9,-1.3] | -3.0 [-4.1,-2.0] | 0.116 | **0.2 [-0.3,0.9]** | 0.2 [-0.2,0.8] | 0.1 [-0.5,0.9] | 0.336 | 0.2 [-0.2,1.0] | 0.1 [-0.5,0.8] | 0.304 |
| Oedema present | 177 (67.3%) | 138 (68.3%) | 39 (63.9%) | 0.523 | 88 (66.7%) | 89 (67.9%) | 0.826 | **-** | - | - |  | - | - |  |
| Days in hospital | 11.0 [8.0,16.0] | **10.0 [7.0,14.0]** | **15.0 [9.0,23.0]** | **<0.001** | **10.0 [7.0,14.0]** | **13.0 [8.0,18.0]** | **0.017** | **-** | **-** | **-** |  | **-** | **-** |  |
| Diarrhoea present | 129 (48.9%) | 100 (49.5%) | 29 (46.8%) | 0.707 | 67 (50.8%) | 62 (47.0%) | 0.538 | **-** | - | - |  | - | - |  |
| Pneumonia present | 22 (9.2%) | 14 (7.7%) | 8 (13.8%) | 0.161 | 8 (6.7%) | 14 (11.6%) | 0.193 | **-** | - | - |  | - | - |  |
| Sepsis present | 11 (4.6%) | 8 (4.4%) | 3 (5.2%) | 0.805 | 5 (4.2%) | 6 (5.0%) | 0.779 | **-** | - | - |  | - | - |  |
| Died | 28 (11.2%) | **10 (5.0%)** | **18 (37.5%)** | **<0.001** | **2 (1.5%)** | **26 (22.0%)** | **<0.001** | **-** | **-** | **-** |  | **-** | **-** |  |
| Poor outcome over 48 weeks^1^ | 45 (18.7%) | **32 (15.8%)** | **13 (33.3%)** | **0.010** | 19 (14.4%) | 26 (23.9%) | 0.061 | **-** |  |  |  |  |  |  |
| LPS/LMR result also present | 202 (76.5%) | 127 (62.9%) | **5 (8.1%)** | **<0.001** | **127 (96.2%)** | **75 (56.8%)** | **<0.001** | **-** | 81 (82.7%) | 12 (16.0%) |  | 81 (87.1%) | **17 (21.2%)** |  |
| Number of timepoints of LMR/LPS per patient (mean) |  | 2.54 |  |  | 1.67 |  |  |  | 1.00 |  |  | 1.00 |  |  |
| Total samples (n) |  | 513 |  |  | 221 |  |  |  | 98 |  |  | 93 |  |  |

Supplementary Table 2: Full details of children with and without samples

LMR – lactulose mannitol ratio; LPS – lipopolysaccharide; WHZ – weight for height z-score.

|  | **Urinary Lactulose**  **(μg/mL)**  **Log_10_** | **Urinary Mannitol**  **(μg/mL)**  **Log_10_** | **Lactulose:**  **Mannitol ratio**  **Log_10_** | **LPS level**  **(EU/mL)**  **Log_10_** |
| --- | --- | --- | --- | --- |
| Unadjusted |  |  |  |  |
| Controls |  |  |  |  |
| Level | -0.50 | 0.11 | -0.62 | 0.09 |
| 95% CI | (-0.70,-0.30) | (-0.10, 0.33) | (-0.79,-0.45) | (-0.12, 0.30) |
| p-value | <0.001 | 0.310 | <0.001 | 0.391 |
| Adjusted^1^ |  |  |  |  |
| Controls |  |  |  |  |
| Level | -0.32 | 0.054 | -0.39 | 0.11 |
| 95% CI | (-0.62,-0.02) | (-0.27, 0.38) | (-0.65,-0.14) | (-0.20, 0.42) |
| p-value | 0.037 | 0.741 | 0.002 | 0.468 |
| ^1^variables adjusted for: HIV, oedema, diarrhoea, whz, country, age, breast feeding status | | | | |

Supplementary Table 3: Longitudinal modelling of LPS and LMR results showing changes over the controls

Graphical representation of the results is shown in Figure 2, with the adjusted results shown in Supplementary Figure 1. Analysed using mixed effects modelling with the participant ID as the random effect, and the variables stated as the fixed effect. Results were log_10_-transforned prior to analysis. CI – confidence interval; LMR – lactulose mannitol ratio; LPS – lipopolysaccharide.

| **Log_10_ biomarker level**  **(pg/mL unless stated)** | **For each log_10_ LPS rise** | **95% CI** | **p-value** | **Adj P-value** | **Obs** |
| --- | --- | --- | --- | --- | --- |
| CRP (mg/L) | **0.154** | **(0.040,0.267)** | **0.008** | **0.038** | **n=511** |
| CD-163 (ng/mL) | 0.032 | (-0.012,0.076) | 0.157 | 0.411 | n=511 |
| LBP (ng/mL) | 0.037 | (-0.012,0.085) | 0.138 | 0.391 | n=508 |
| CD14 | 0.020 | (-0.009,0.049) | 0.180 | 0.438 | n=510 |
| **TNF-α** | **0.031** | **(0.017,0.045)** | **<0.001** | **<0.001** | **n=502** |
| **IL-6** | **0.091** | **(0.064,0.118)** | **<0.001** | **<0.001** | **n=502** |
| IL-33 | -0.002 | (-0.018,0.015) | 0.850 | 0.885 | n=502 |
| IL-1β | 0.003 | (-0.011,0.017) | 0.671 | 0.877 | n=502 |
| Interferon-γ | 0.004 | (-0.011,0.019) | 0.626 | 0.877 | n=502 |
| **IL-1ra** | **0.100** | **(0.065,0.135)** | **<0.001** | **<0.001** | **n=502** |
| **CCL3** | **0.063** | **(0.041,0.086)** | **<0.001** | **<0.001** | **n=502** |
| **CCL4** | **0.030** | **(0.020,0.040)** | **<0.001** | **<0.001** | **n=502** |
| **IL-8** | **0.158** | **(0.122,0.195)** | **<0.001** | **<0.001** | **n=502** |
| IL-2 | 0.015 | (-0.004,0.034) | 0.133 | 0.391 | n=502 |
| IL-10 | 0.023 | (0.003,0.042) | 0.021 | 0.088 | n=502 |
| D-dimer | 0.013 | (-0.042,0.067) | 0.653 | 0.877 | n=502 |
| L-selectin | 0.009 | (-0.014,0.033) | 0.444 | 0.731 | n=502 |
| P-selectin | -0.003 | (-0.022,0.017) | 0.786 | 0.885 | n=502 |
| Thrombopoietin | 0.005 | (-0.008,0.018) | 0.452 | 0.731 | n=502 |
| VCAM-1 | 0.046 | (-0.002,0.093) | 0.061 | 0.207 | n=502 |
| ICAM-1 | 0.023 | (-0.036,0.083) | 0.442 | 0.731 | n=502 |
| Eotaxin | 0.009 | (-0.016,0.033) | 0.489 | 0.756 | n=502 |
| EGF | -0.025 | (-0.067,0.017) | 0.242 | 0.513 | n=502 |
| VEGF | -0.014 | (-0.046,0.019) | 0.417 | 0.731 | n=502 |
| PlGF | 0.006 | (-0.016,0.028) | 0.590 | 0.873 | n=502 |
| Angiopoietin | 0.018 | (-0.027,0.064) | 0.433 | 0.731 | n=502 |
| GCSF | 0.008 | (-0.035,0.051) | 0.712 | 0.885 | n=502 |
| GM-CSF | -0.008 | (-0.119,0.103) | 0.885 | 0.885 | n=502 |
| IGFBP-3 | -0.035 | (-0.091,0.021) | 0.215 | 0.487 | n=500 |
| α1-AT (mg/mL) | -0.012 | (-0.091,0.066) | 0.758 | 0.885 | n=303 |
| Neopterin (nmol/L) | 0.121 | (0.003,0.239) | 0.044 | 0.167 | n=296 |
| MPO (ng/mL) | 0.030 | (-0.145,0.204) | 0.739 | 0.885 | n=318 |
| GLP-2 (ng/mL) | -0.009 | (-0.110,0.092) | 0.861 | 0.885 | n=474 |
| IFABP | -0.006 | (-0.063,0.051) | 0.830 | 0.885 | n=510 |
|  | | | | | |

Supplementary Table 4: Changes in biomarker level for each log_10_ increase in plasma LPS concentration

Samples are from children with SAM at all timepoints. *** Adj. p-value<0.00, ** Adj. p-value<0.01, * Adj. p-value<0.05. Variables adjusted for: HIV, oedema, diarrhoea, whz, country, age, breast feeding status. ^1^Adjusted P-value adjusted for false discovery by Benjamini-Hochberg false-discovery rate correction. LPS – lipopolysaccharide.

| **Log_10_ biomarker level**  **(pg/mL unless stated)** | **Each log_10_ LMR increase** | **95% CI** | **p-value** | **Adj P-value^1^** | **Obs** |
| --- | --- | --- | --- | --- | --- |
| CRP (mg/L) | 0.191 | (-0.072,0.454) | 0.155 | 0.384 | n=219 |
| CD-163 (ng/mL) | -0.044 | (-0.106,0.017) | 0.158 | 0.384 | n=219 |
| LBP (ng/mL) | 0.020 | (-0.060,0.100) | 0.619 | 0.782 | n=218 |
| CD14 | -0.022 | (-0.088,0.044) | 0.512 | 0.782 | n=219 |
| TNF-α | -0.022 | (-0.052,0.008) | 0.156 | 0.384 | n=213 |
| IL-6 | 0.004 | (-0.043,0.050) | 0.881 | 0.907 | n=213 |
| IL-33 | -0.045 | (-0.085,-0.006) | 0.026 | 0.139 | n=213 |
| IL-1β | -0.037 | (-0.068,-0.006) | 0.021 | 0.139 | n=213 |
| Interferon-γ | -0.023 | (-0.056,0.010) | 0.171 | 0.388 | n=213 |
| IL-1ra | 0.072 | (0.008,0.136) | 0.027 | 0.139 | n=213 |
| CCL3 | 0.045 | (-0.007,0.098) | 0.089 | 0.377 | n=213 |
| CCL4 | 0.005 | (-0.015,0.024) | 0.628 | 0.782 | n=213 |
| IL-8 | 0.021 | (-0.048,0.090) | 0.556 | 0.782 | n=213 |
| IL-2 | -0.028 | (-0.069,0.014) | 0.194 | 0.413 | n=213 |
| IL-10 | 0.009 | (-0.023,0.041) | 0.582 | 0.782 | n=213 |
| D-dimer | -0.011 | (-0.132,0.109) | 0.855 | 0.907 | n=213 |
| **L-selectin** | **-0.068** | **(-0.109,-0.028)** | **0.001** | **0.018** | **n=213** |
| P-selectin | -0.005 | (-0.048,0.038) | 0.824 | 0.904 | n=213 |
| Thrombopoietin | -0.023 | (-0.053,0.008) | 0.140 | 0.384 | n=213 |
| VCAM-1 | -0.026 | (-0.138,0.086) | 0.647 | 0.782 | n=213 |
| ICAM-1 | -0.006 | (-0.133,0.120) | 0.921 | 0.921 | n=213 |
| Eotaxin | -0.012 | (-0.067,0.043) | 0.667 | 0.782 | n=213 |
| EGF | -0.070 | (-0.165,0.024) | 0.145 | 0.384 | n=213 |
| VEGF | -0.039 | (-0.113,0.034) | 0.291 | 0.522 | n=213 |
| PlGF | -0.025 | (-0.077,0.027) | 0.336 | 0.571 | n=213 |
| Angiopoietin | -0.013 | (-0.107,0.082) | 0.790 | 0.895 | n=213 |
| GCSF | 0.030 | (-0.066,0.126) | 0.537 | 0.782 | n=213 |
| GM-CSF | -0.097 | (-0.350,0.156) | 0.452 | 0.731 | n=213 |
| IGFBP-3 | -0.081 | (-0.221,0.060) | 0.258 | 0.487 | n=212 |
| α1-AT (mg/mL) | -0.068 | (-0.188,0.051) | 0.258 | 0.487 | n=172 |
| Neopterin (nmol/L) | 0.128 | (-0.048,0.303) | 0.153 | 0.384 | n=173 |
| MPO (ng/mL) | 0.361 | (0.038,0.684) | 0.029 | 0.139 | n=177 |
| **GLP-2 (ng/mL)** | **0.383** | **(0.160,0.606)** | **<0.001** | **0.018** | **n=209** |
| IFABP | 0.135 | (0.039,0.231) | 0.006 | 0.069 | n=219 |

Supplementary Table 5: Changes in biomarker level for each log_10_ increase in lactulose:mannitol ratio

Samples are from children with SAM at all timepoints. *** Adj. p-value<0.001, ** Adj. p-value<0.01, * Adj. p-value<0.05. Variables adjusted for: HIV, oedema, diarrhoea, whz, country, age, breast feeding status

^1^Adjusted P-value adjusted for false discovery by Benjamini-Hochberg correction. LMR – lactulose mannitol ratio.

|  | | **Unadjusted** | | | **Numbers: Failures/ total n** |
| --- | --- | --- | --- | --- | --- |
|  | | **Hazard Ratio** | **95% CI** | **p-value** |  |
| Baseline LMR level (log_10_) | | **5.73** | **(1.03,32.0)** | **0.046** | 5/36 |
| Baseline LPS level (EU/mL log_10_) | | 1.17 | (0.71, 1.90) | 0.538 | 18/120 |
|  |  |  |  |  |  |

Supplementary Table 6: Results of effect of baseline levels of LMR and LPS on death or hospital readmission

Analysis restricted to baseline biomarker values. LMR – lactulose mannitol ratio; LPS – lipopolysaccharide

|  |  | **Lactulose:mannitol ratio** | | | **LPS levels** | | |  |
| --- | --- | --- | --- | --- | --- | --- | --- | --- |
|  |  | **LMR 10th centile** | **LMR** | **LMR 90th centile** | **LPS 10th centile** | **LPS level** | **LPS 90th centile** |  |
| **All values** | Controls |  |  |  |  |  |  |  |
|  | Obs | n=93 | n=93 | n=93 | n=98 | n=98 | n=98 |  |
|  | median | -1.39 | -1.39 | -1.39 | -2.85 | -2.85 | -2.85 |  |
|  | IQR | (-1.61,-1.13) | (-1.61,-1.13) | (-1.61,-1.13) | (-2.85,-2.05) | (-2.85,-2.05) | (-2.85,-2.05) |  |
|  | SAM children |  |  |  |  |  |  |  |
|  | Obs | n=890 | n=220 | n=890 | n=902 | n=513 | n=902 |  |
|  | median | -1.48 | -1.16 | -0.44 | -2.85 | -2.85 | -2.00 |  |
|  | IQR | (-1.62,-1.27) | (-1.45,-0.84) | (-0.84,-0.04) | (-2.85,-2.85) | (-2.85,-2.40) | (-2.85,-1.41) |  |
| **Values at each timepoint** | Baseline |  |  |  |  |  |  |  |
|  | Obs | n=224 | n=37 | n=224 | n=227 | n=126 | n=227 |  |
|  | median | -1.27 | -0.75 | -0.04 | -2.85 | -2.85 | -1.07 |  |
|  | IQR | (-1.27,-1.27) | (-1.07,-0.39) | (-0.04,-0.04) | (-2.85,-2.70) | (-2.85,-2.05) | (-2.85,-1.07) |  |
|  | t-test over controls | <0.001 | <0.001 | <0.001 | 0.002 | 0.504 | <0.001 |  |
|  | Discharge |  |  |  |  |  |  |  |
|  | Obs | n=222 | n=12 | n=222 | n=229 | n=121 | n=229 |  |
|  | median | -1.48 | -0.71 | 0.02 | -2.85 | -2.85 | -1.41 |  |
|  | IQR | (-1.48,-1.48) | (-1.00,-0.29) | (0.02, 0.02) | (-2.85,-2.85) | (-2.85,-2.40) | (-2.85,-1.41) |  |
|  | t-test over controls | 0.003 | <0.001 | <0.001 | <0.001 | 0.096 | 0.007 |  |
|  | 12 weeks |  |  |  |  |  |  |  |
|  | Obs | n=222 | n=77 | n=222 | n=224 | n=123 | n=224 |  |
|  | median | -1.62 | -1.21 | -0.44 | -2.85 | -2.85 | -1.74 |  |
|  | IQR | (-1.62,-1.43) | (-1.46,-0.92) | (-0.95,-0.44) | (-2.85,-2.85) | (-2.85,-2.70) | (-2.85,-1.74) |  |
|  | t-test over controls | 0.011 | 0.005 | <0.001 | <0.001 | 0.012 | 0.518 |  |
|  | 48 weeks |  |  |  |  |  |  |  |
|  | Obs | n=222 | n=94 | n=222 | n=222 | n=143 | n=222 |  |
|  | median | -1.74 | -1.31 | -0.84 | -2.85 | -2.85 | -2.52 |  |
|  | IQR | (-1.74,-1.38) | (-1.50,-1.08) | (-1.20,-0.84) | (-2.85,-2.85) | (-2.85,-2.70) | (-2.85,-2.00) |  |
|  | t-test over controls | <0.001 | 0.335 | <0.001 | <0.001 | 0.002 | 0.137 |  |
| **Longitudinal** | Unadjusted Control Level | -0.15 | -0.62 | -1.18 | 0.32 | 0.09 | -0.47 |  |
|  | 95% CI | (-0.23,-0.07) | (-0.79,-0.45) | (-1.26,-1.10) | (0.16, 0.47) | (-0.12, 0.30) | (-0.66,-0.29) |  |
|  | p-value | <0.001 | <0.001 | <0.001 | <0.001 | 0.391 | <0.001 |  |
|  | Adjusted Control Level^1^ | -0.16 | -0.39 | -0.96 | 0.19 | 0.11 | -0.07 |  |
|  | 95% CI | (-0.30,-0.03) | (-0.65,-0.14) | (-1.09,-0.82) | (-0.08, 0.45) | (-0.20, 0.42) | (-0.37, 0.22) |  |
|  | p-value | 0.017 | 0.002 | <0.001 | 0.165 | 0.468 | 0.622 |  |
| **Survival** | Unadjusted HR | 1.86 | 6.14 | 3.79 | 0.89 | 0.87 | 0.85 |  |
|  | 95% CI | (0.74, 4.70) | (1.03,36.81) | (0.58,24.86) | (0.45, 1.75) | (0.40, 1.88) | (0.33, 2.21) |  |
|  | p-value | 0.190 | 0.047 | 0.164 | 0.733 | 0.718 | 0.742 |  |
|  |  | | | | | | | |

Supplementary Table 7: Sensitivity analysis examining the potential impact of missing data if there were a departure from the missing at random assumption

A sensitivity analysis was carried out by replacing the missing values with a low value set at the 10^th^ centile of the available data at that timepoint, and a high value set at the 90^th^ centile. The analyses were re-run to see whether these new levels had an impact on the overall conclusion of the analysis. ^1^variables adjusted for: HIV, oedema, any diarrhoea, WHZ, country, age, and breastfeeding status. CI – confidence interval; HR – hazard ratio; IQR – interquartile range; LMR – lactulose mannitol ratio; LPS – lipopolysaccharide.

*lactulose recovery* = $\frac{lactulose out}{lactulose in}$ = $\frac{{lactulose}_{conc. urine} \times urine volume}{2 \times250mg \times weight (kg)}$

*mannitol recovery* = $\frac{mannitol out}{mannitol in}$ = $\frac{{mannitol}_{conc. urine} \times urine volume}{2 \times50mg \times weight (kg)}$

LMR $=\frac{lactulose recovery}{mannitol recovery}$ = $\frac{\frac{{lactulose}_{conc. urine} \times urine volume}{2 \times250mg \times weight (kg)}}{\frac{{mannitol}_{conc. urine} \times urine volume}{2 \times50mg \times weight (kg)}}$ = $\frac{{lactulose}_{conc. urine}}{{5 \times mannitol}_{conc. urine}}$

Supplementary Table 8: Derivation of the lactulose:mannitol ratio equation

Derivation of the lactulose:mannitol ratio (LMR) equation used. This requires the measured urinary concentration of lactulose, and the measured urinary concentration of mannitol.

The administered lactulose mannitol solution contained 250mg of lactulose and 50mg of mannitol per millilitre, and was given at 2mL/kg, up to a maximum of 20mL.

Supplementary Figure 1: Adjusted results for changes over time in children with SAM compared with adequately nourished controls for A) LPS level and B) LMR

Mixed effects modelling with the patient ID as the random effect, and HIV, country, oedema, diarrhoea, WHZ, age, and breastfeeding as the fixed effect. Total numbers at each timepoint are shown beneath the timepoint, with the number below limit of detection (blod) shown beneath. The blod values in the LPS level are included in the analysis at the level of detection divided by √2.

Supplementary Figure 2: Results for LPS analysis, with the out-of-range (OOR) results excluded, both A) unadjusted, and B) adjusted for variables shown

This does not substantially alter the results excluding the results which are OOR (out-of-range) below the limit of detection. 95% confidence intervals are the result of the mixed effects modelling with the patient ID as the random effect, and HIV, country, oedema, diarrhoea, WHZ, age, and breastfeeding as the fixed effect.

Supplementary Figure 3: Graphs showing the correlation between a) LMR and LPS level, b) Urinary lactulose concentration and LPS level, and c) urinary mannitol concentration and LPS levels

Supplementary Figure 4: Timing of LM urinary testing compared with baseline HOPE-SAM visit

LM – lactulose:mannitol.
